# From admiration to retribution: the mediating role of nurses’ vengeful behaviors in the link between nurse managers’ narcissism and nurses’ counterproductive work behaviors

**DOI:** 10.1186/s12912-025-03308-1

**Published:** 2025-07-04

**Authors:** Amal Diab Ghanem Atalla, Loujain Saud Sharif, Alaa Mahsoon, Maram Banakhar, Amira Mohammed Ali, Samia Mohamed Sobhi Mohamed

**Affiliations:** 1 Faculty of Nursing, Department of Nursing Administration, Alexandria University, Alexandria, Egypt; 2 Faculty of Nursing, Psychiatric and Mental Health Nursing Department, King Abdulaziz University, Jeddah, Saudi Arabia; 3 Faculty of Nursing, Public Health Department, King Abdulaziz University, Jeddah, Saudi Arabia; 4 Faculty of Nursing, Department of Psychiatric Nursing and Mental Health, Alexandria University, Alexandria, Egypt

**Keywords:** Vengeful behaviors, Narcissism, Counterproductive work behaviors, Nurse, Manager, And healthcare organization

## Abstract

**Background:**

Constructive works by nurse managers are thought to affect nurses’ profession, satisfaction with work, and engagement with the healthcare organization. instead, it has been shown that the negative behavior of nurse supervisors, such as narcissism, has impacted the psychological health of the nurses and caused them to engage in counterproductive behavior (CWB). Hence, this study aims to determine the influence of the mediating role of nurses’ vengeful behaviors in the link between nurse managers’ narcissism and nurses’ counterproductive work behaviors.

**Methods:**

A descriptive correlational study was conducted in an Egyptian hospital. A convenient sample of staff nurses (*N* = 400) who agreed to participate in the study answered the narcissistic admiration and rivalry questionnaire (NARQ), counterproductive work behaviors, and nurses’ counterproductive work behaviors questionnaires, which were proven to be valid and reliable study measures. Descriptive and inferential statistics were applied, and relationships were presented using a path model.

**Ethical Considerations:**

Ethics Committee approval, written informed consent, data privacy and confidentiality, and participants’ rights to voluntary participation and withdrawal were maintained.

**Results:**

Vengeful behaviors (B = 0.191, β = 0.137, t = 2.768, *p* < 0.001) and unproductive work behaviors (B = 0.099, β = 0.231, t = 4.654, *p* < 0.001) were substantially predicted by narcissistic adoration and rivalry. Of the variance in CWBs, 8.4% was explained by the model. Vengeful behaviors’ mediating role was validated by path analysis, and the model fit the data well (e.g., CFI = 0.95, RMSEA = 0.04).

**Conclusion:**

The narcissism of nurse supervisors fuels the retaliatory and ineffective actions of staff nurses. Reducing toxic leadership attributes may improve corporate outcomes and decrease workplace deviance.

**Nursing implications:**

Nurse supervisors should receive training in emotional intelligence and ethical leadership from healthcare institutions. The quality of patient treatment and worker satisfaction may both be improved by fostering a friendly and open work environment. Future studies should examine long-term effects and evaluate strategies to lessen negative leadership traits.

**Clinical trial number:**

Not applicable.

## Background

In nursing leadership research, nurse managers’ positive actions are considered to have an impact on nurses’ job satisfaction, engagement with the healthcare organization, and profession [1]. Otherwise, it has been noticed that the negative conduct of nurse managers, including narcissism, has affected the psychological well-being of the nurses and led to counterproductive behavior (CWB) among them. Thus, it is necessary to investigate how nurse managers behave in their work-life balance and how their positive or negative personality traits affect these actions. One of the most studied negative personality traits is narcissism [1, 2].

Narcissism is “a complex personality traits and processes that involve a grandiose yet fragile sense of self as well as a preoccupation with success and demands for admiration” [3]. Nurses and healthcare organizations are negatively impacted by narcissistic behavior. Frequent absences, low job satisfaction, poor job performance, low productivity, low work enthusiasm, job anxiety and burnout, frustration, disappointment, gossip, weariness, and increased nurse turnover are all possible outcomes for nurses [4]. At the healthcare organizational level, it results in problems with safety, the quality of the work environment, and the healthcare organization. Additionally, it encourages the growth of a toxic corporate culture, which may eventually result in the emergence of more toxic leaders [5].

The performance of the healthcare organization and the well-being of its nurses are seriously threatened by counterproductive work behavior (CWB), which is pervasive in the workplace. Narcissism is a possible risk factor for CWB [6]. Nurses who act contrary to an organization’s fundamental objectives are engaging in counterproductive work behavior (CWB). Another clear definition is a planned unwanted activity that could harm the healthcare organization [7]. CWB results in regrettable acts, time and resource waste, and the destruction of belongings, among other things. According to Karatuna et al. (2020), it has a significant negative impact on societies and their workers [8].

In the workplace, vengeful behaviors are ineffective, punishing, and destructive responses to perceived misconduct [9, 10]. vengeful actions have a variety of negative and/or good effects, academic publications often highlight the negative effects of feeling unfairly treated. In this regard, retaliation can lead to counterproductive behavior, protracted conflicts and is frequently linked to several detrimental psychological effects [11]. Furthermore, employees’ perceptions of abusive managers frequently result in reprisal behaviors and harsh judgments of those managers [12].

There is little empirical data on how nurse managers’ narcissistic features explicitly affect nurses’ counterproductive work behaviors (CWBs) in healthcare settings, even though previous research has examined the consequences of toxic leadership on employee outcomes. Without looking at vindictive behavior as a mediating mechanism, most of the previous research has concentrated on general workplace aggression or exhaustion. This study fills a crucial vacuum since clinical settings are high-stakes affairs where poor leadership can jeopardize patient safety, nurse retention, and treatment quality. The study offers fresh insights into the psychological mechanisms via which leadership narcissism may worsen workplace conduct in nursing by examining the mediation role of vindictive behaviors. The results can guide focused leadership initiatives to improve patient care outcomes and foster healthier organizational cultures.

## Literature review

### Leader’ narcissism

An inflated self-perception, a strong sense of psychological superiority and entitlement, and a lack of empathy are characteristics of narcissism [13]. Narcissism is very common in hospitals, particularly among leaders [14]. It is, therefore, unavoidable that nurses would contact narcissistic bosses. Nurses are expected to engage in more extra-role behaviors by narcissistic leaders because they are self-centered [15].

According to Zeng (2020), narcissism is characterized by a complex set of psychological traits and processes, including a grandiose yet frail sense of self, obsession with success, and demands for adulation [3]. One type of leadership is narcissistic leadership, when the leader’s primary focus is on projecting himself and shows no concern for his followers [16]. According to Disque (2020), a narcissistic leader is driven by power and the admiration of their followers and who holds grandiose views [17]. Grandiosity, self-absorption, vanity, entitlement, tendency to take advantage of others, and excessively positive self-perceptions are characteristics of those leaders [18].

13.6% of staff in the US are subjected to abusive monitoring, according to the study, so this has a significant financial impact on organizations due to absenteeism, attrition, and poor performance. Since the 1980s, individuals have been interested in the negative aspects of leadership. Due to focusing on the negative aspects of leadership, several academics have highlighted how narcissism affects interactions between managers and their subordinates. Additionally, narcissism is expressly included in the so-called dark triad of personality [15].

Because narcissistic leaders have a propensity to enhance their image at the expense of others to increase their power, narcissism can lead to short-term success but ultimately has detrimental effects on followers’ professional development, well-being, and quality of work. Narcissists do not form enduring, healthy relationships and are indifferent to the welfare of others, even yet they require affirmation from their surroundings and praise [19].

### Nurses’ counterproductive work behaviors

Deviant conduct is one of the most prevalent problems at work. Examples of actions that mainly transgress moral and ethical norms include sabotage, animosity, and verbal or physical assault [20]. “Counterproductive job practices” or “counterproductive work behaviors” (CWB) are the general term for all these behaviors [21]. Among nurses, counterproductive work behaviors (CWBs) include deliberate acts that cause harm to organizations or their members, such as destruction, workplace rudeness, and absenteeism [21]. Research has identified several factors that contribute to CWBs in nursing. Abuse of supervision, characterized by aggressive verbal and nonverbal behaviors from superiors, has been linked to higher CWBs among nurses. This link is mediated by perceptions of unfairness, while it is moderated by individual differences such as locus of control and power distance orientation [2, 22].

CWBs are also influenced by personality factors. CWBs aimed at both individuals and organizations have a favorable correlation with moral disengagement, which is the cognitive process of rationalizing unethical behavior. A strong moral identity, on the other hand, can counteract the impact of moral disengagement on CWBs, emphasizing the significance of an ethical self-concept in professional behavior [23, 24]. Increasing CWBs has been linked to organizational issues such as workplace stress and overload. This relationship can be made worse by perceived unfairness, which emphasizes the necessity of just and encouraging work settings [25].

Bullying and rudeness among coworkers are examples of interpersonal dynamics that greatly contribute to CWBs. Nurses may experience stress, burnout, and retaliatory actions as a result of peer rudeness and hierarchical aggression in which supervisors abuse their staff. Additionally, CWBs have been linked to organizational restrictions, proactive coping, and autonomy; hence, increasing nurses’ authority over their work and providing them with sufficient resources may lessen these behaviors [23, 26].

CWBs among nurses are complex phenomena that are impacted by organizational factors, interpersonal connections at work, supervisory conduct, and individual personality features. Promoting moral principles, guaranteeing equitable treatment, encouraging constructive interpersonal relationships, and skillfully handling work-related pressures are all essential components of a complete strategy to address these challenges [2, 23–26].

### Nurses’ vengeful behaviors

Nurses’ vengeful behaviors, which are defined as acts meant to inflict revenge or cause harm to colleagues, are attracting attention because of the negative effects they have on the staff, which lead to impaired staff retention, upset staff moral condition, and decreased team cohesion. Moreover, it impairs the quality of patient care and generally negatively affects the healthcare settings. These acts can take the form of bullying, horizontal aggression, or rudeness in the workplace [27].

Numerous antecedents to the emergence of this type of behavior, such as violence, abuse, and toxic management, have been the focus of recent studies. For example, Koç et al. (2022) investigated the association between nurses’ vengeful behaviors and toxic management and found that exposure to toxic management is a significant antecedent of these behaviors. Remarkably, the study did not find any statistical evidence that psychological well-being moderates this association, indicating that even nurses who have excellent psychological well-being are vulnerable to the harmful effects of toxic management [27, 28].

### Theoretical underpinning

In this study the four theoretical frameworks integrate psychological and organizational behavior theories to explain these relationships which are social exchange theory, moral disengagement theory, affective events theory (AET) and psychological contract theory (PCT) which collectively could explain how nurses’ vengeful behaviors in the link between nurse managers’ narcissism and nurses’ counterproductive work behaviors.

Social Exchange Theory (SET), which was established by Blau (1964) and Homans (1958), describes social interactions as reciprocal exchanges in which people aim to maximize relationship advantages and minimize relationship costs. According to SET, when workers believe they are being treated fairly, they react positively; yet, when they are mistreated, they could react negatively in return. A negative interaction with nurses might result from narcissistic nurse supervisors’ manipulative, exploitative, or self-centered actions. Furthermore, in retaliation for perceived injustice, nurses may engage in vindictive actions, which can lead to counterproductive work behaviors (CWBs) [29, 30].

The Social Exchange Theory (SET) offers an engaging prism through which to view how beliefs of reciprocity and fairness influence employees’ conduct in corporate contexts. According to SET, relationships at work are based on reciprocal exchanges, in which people react positively to the treatment they receive and negatively to perceived abuse. In the healthcare setting, nurses may see their nurse managers’ self-centered, exploitative, or manipulative behaviors as a breach of relational fairness and respond with retaliation if they believe the managers are narcissistic. These reactions frequently take the form of vindictive actions, as nurses deliberately try to “get even” or reestablish the relationship’s perceived equilibrium. These acts of retaliation can eventually develop into more general counterproductive work behaviors (CWBs), such as purposeful rule violating, compromising teamwork, or withholding effort. According to SET, spiteful conduct is therefore a behavioral and psychological mechanism that converts perceived managerial narcissism into CWBs [29, 30].

The authors use three auxiliary theories to support psychological dynamics in this process, even though SET serves as the main explanatory basis. According to Bandura’s (1999) Moral Disengagement Theory, nurses may rationalize retaliatory actions as ethically acceptable reactions to unjust treatment. Anger or resentment are examples of emotional reactions that might feed vengeful intentions, according to the Affective Events Theory (AET) (Weiss & Cropanzano, 1996). Finally, narcissistic leadership may be interpreted as a violation of implicit expectations, which would further incite retaliatory reactions, according to Psychological Contract Theory (PCT) (Rousseau, 1989).

Theory of moral disengagement: According to Bandura (1999), individuals disengage from moral standards to excuse unethical behavior. For example, when nurses are mistreated by narcissistic managers, they may engage in retaliatory activities (such as sabotage and withholding effort) by viewing them as just punishment rather than immorality [31].

According to Weiss & Cropanzano’s (1996) Affective Events Theory (AET), emotions at work influence behavior. When nurses are exposed to narcissistic leadership, they may feel angry or resentful, which can lead to vindictive actions that become CWBs. Finally, the theory of the psychological contract (PCT), according to Rousseau (1989), states that workers expect managers and employers to treat them fairly. These expectations are broken by narcissistic managers, which causes nurses to respond to contract breaches by engaging in vengeful behaviors and CWBs [32, 33].

By incorporating these perspectives under the combined framework of Social Exchange Theory, this study positions vengeful behavior as a key mediator in the relationship between nurse managers’ narcissism and nurses’ CWBs.

### Hypothesis

From the previous conceptualizations, the following hypotheses were proposed for this study:

#### H1

There is a significant relationship between nurse managers’ narcissism and nurses’ counterproductive work behaviors.

#### H2

Nurses’ vengeful behaviors mediate the relationship between the nurse managers’ narcissism and nurses’ counterproductive work behaviors.

**Fig. 1:**
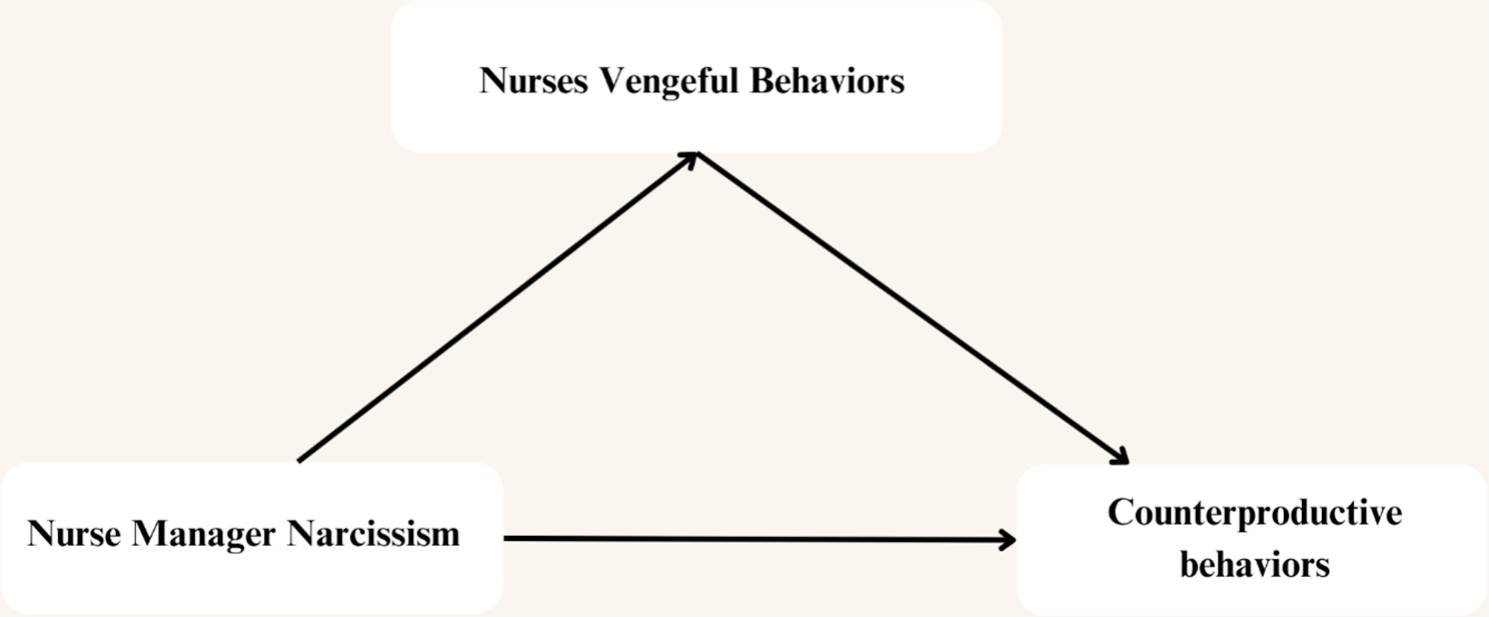
proposed conceptual framework by researcher

From the conceptualizations, the authors planned a conceptual model for this study (Fig. 1), assuming that nurse managers’ narcissism is the independent variable, nurses’ counterproductive work behaviors are the dependent variable, and nurses’ vengeful behaviors act as a mediator.

### Significance of study

As it addresses the role of mediating nurses’ vengeful behaviors in the association between nurses’ counterproductive work behaviors (CWBs) and nurse managers’ narcissism, this study is noteworthy. Gaining an understanding of this dynamic is essential for promoting better working relationships, enhancing patient care, and guaranteeing efficient nursing administration. Organizational psychology has extensively studied narcissistic leadership. Its effects on healthcare, especially nursing, are still not well understood [34, 35]. Additionally, this study emphasizes how narcissistic management promotes CWBs through the psychological mechanism of vengeful behaviors. Through a combination of management, emotional responses, and workplace deviance, this study offers a thorough understanding of how toxic managerial practices indirectly result in detrimental organizational results [36].

Essentially, hospital administrators and policymakers might apply the study’s findings to guide the creation of interventions aimed at lessening the negative consequences of narcissistic management. Healthcare organizations might set in place management training programs targeted at encouraging more encouraging and emotionally intelligent managerial practices by determining how this type of management leads to vengeful behaviors and, thus, CWBs. Additionally, resolving these workplace concerns can greatly increase nurses’ job satisfaction, cut stress levels, and decrease burnout rates, all of which will improve the general well-being of the workforce [37]. The findings of this research might affect personnel mental health support, resolution of conflicts, and leader selection criteria, which could result in a more efficient and long-lasting healthcare system. This study is important because national healthcare policies, such as those promoted by the World Health Organization (WHO) and the American Nurses Association (ANA), stress the value of effective management and workplace well-being [38]. Hence, this study aims to determine the influence of the mediating role of nurses’ vengeful behaviors in the link between nurse managers’ narcissism and nurses’ counterproductive work behaviors.

## Methods

### Study design

This study used a cross-sectional, descriptive correlational design in compliance with the STROBE standards.

### Study setting

This study will be carried out using a descriptive correlational research approach. Alexandria Main University Hospital, which is connected to Alexandria University, will be the site of this investigation. With more than 6760 beds, it offers public non-paid health services. It is Alexandria’s biggest educational university hospital. This Egyptian hospital was chosen as the study location because of its vibrant and diverse healthcare system, which greatly influences workplace results through nurse-manager relationships. The manifestation of narcissism and vindictive behaviors in Egyptian healthcare settings may be influenced by cultural factors. The impacts of management narcissism may be exacerbated by the hierarchical structure that is typical in hospitals. Examining this setting provides important insights into ineffective work practices in a Middle Eastern cultural setting.

All 23 critical care units will be used for the study, including the second, third, and fourth units; medical emergency units; surgical emergency units; intensive care units of emergency operations; transitional intensive care units; new transitional units; intensive care units for continuous renal replacement therapy and critical cases; toxicity units; Maxillo Facial and Plastic Surgery intensive care units; burn intensive care units; pulmonology intensive care units; neurosurgery intensive care units (1) and (2); pediatric neurosurgery intensive care units; hematemesis ICU; urology ICU, Anesthesia ICU, Systemic Lupus ICU, Hepatic Transitional ICU, Diabetics ICU, and Ear, Nose, and Trachea (ENT ICU).

### Participants

Participants were chosen based on their availability and accessibility using a convenience sampling technique, which is a form of non-probability sampling. A total of 400 staff nurses were recruited for the study, which was directed at all qualified nurses employed by a single hospital in Egypt. According to the inclusion criteria, participants had to: (1) be actively involved in direct patient care; and (2) have worked in their present hospital unit for at least a year. Excluded were nurses with less than a year of unit experience or those working in administrative positions. To guarantee familiarity with the hospital’s system, policies, and practices, the sample was chosen. We recognize that the necessary sample size was determined using the Centers for Disease Control and Prevention’s (CDC) Epi Info Stat Calc, which assumes simple random sampling and is based on a 95% confidence level, 5% margin of error, and 50% predicted frequency. The real margin of error, however, recalculates to about 3.8% given an estimated target population of about 1,000 hospital nurses. This demonstrates that, in this institutional setting, the sample size (*n* = 400) is sufficient for generating trustworthy descriptive and inferential statistics.

### Ethical considerations

The research protocol was authorized by the Alexandria University Research Ethics Committee (REC) with reference code number AU-20-8-322 in December 2024, which is a division of the College of Nursing. Nurses were informed of the aim of the study before providing their signed consent. To safeguard identity and secrecy, a code number was given to each questionnaire. Participants were informed about the purpose, procedures, potential risks, and benefits of the study. Written informed consent was obtained from all participants before their inclusion. The data was only used for research, as was assured by the nurses. The ability to leave the study has been confirmed.

### Tools

#### Three tools will be used in this study as follows

##### Tool (1): narcissistic admiration and rivalry questionnaire (NARQ)

Back et al. (2013) developed this tool. With a Likert-type response scale ranging from 1 (totally disagree) to 6 (absolutely agree), the questionnaire consists of 18 items. The tool is separated into two broad strategies, each of which is further subdivided into three subcomponents, each consisting of three elements. Admiration and rivalry are the general strategies. Grandiosity (e.g., “I am great”), striving for uniqueness (e.g., “I show others how special I am”), and Charming Ness (e.g., “I manage to be the center of attention with my outstanding contributions”) make up the first one. According to Back et al. (2013), rivalry is made up of three main components: (a) devaluation (e.g., “most people won’t achieve anything”), (b) striving for supremacy (e.g., “I secretly take pleasure in the failure of my rivals”), and (c) aggressiveness (e.g., “I often get annoyed when I am criticized”). The version utilized in this study was Doroszuk et al.‘s (2019) Spanish adaptation, which had sufficient Cronbach’s Alpha (α) coefficients. Both for admiration (minimum α = 0.78; maximum α = 0.84) and for rivalry (minimum α = 0.81; maximum α = 0.85) [39].

##### Tool (2): nurses’ vengeful behaviors

The authors of this tool were Coelho et al. (2018). Ten elements comprise this assessment tool, which gauges nurses’ perceptions of vengeful behavior [40]. A 5-point Likert scale, from strongly agree (5) to strongly disagree (1), will be used to score the responses. The total score falls between 10 and 50. Higher ratings suggest that nurses engage in more vengeful behaviors. The internal consistency (Coefficient alpha) value was 0.940 [28].

##### Tool (3) counterproductive work behavior checklist (CWB-C)

This tool was developed by Spector et al. [41]. It consists of 33 items and is divided into five dimensions, namely sabotage (3 items), withdrawal (4 items), production deviance (3 items), theft (5 items), and abuse (18 items). Each item asks for a rating of how frequently a constraint is encountered using a five-point scale ranging from 1 never to 5 every day. The scale had an internal consistency reliability of 88. The overall score ranges from 33 to 165.

Demographic data was collected by researchers, including gender, age, years of nursing experience, years of work unit experience, and educational background.

### Tools validity

After being translated into Arabic and English, the three instruments were adjusted appropriately. Resources for analyzing and evaluating item clarity, question types, and content validity were made available to five experts. To guarantee accuracy and preserve the integrity of the study, their suggestions were taken into consideration. To ensure accuracy, a confirmatory factor analysis was performed on the narcissistic admiration and rivalry questionnaire, nurses’ vengeful behaviors, and nurses’ counterproductive work behaviors scales.

To guarantee linguistic and cultural appropriateness, the three tools utilized in this study underwent stringent translation and validation processes. Independent bilingual experts used a forward-backward translation approach to translate the original versions of the Narcissistic Admiration and Rivalry Questionnaire (NARQ), the Nurses’ Vengeful Behaviors Scale, and the Nurses’ Counterproductive Work Behaviors Scale into Arabic.

To guarantee clarity and relevance, cultural adaptation was accomplished through expert panel evaluations and pilot testing with a representative nursing sample. The NARQ (CFI = 0.96, RMSEA = 0.06, χ²/df = 2.5), Nurses’ Vengeful Behaviors (CFI = 0.95, RMSEA = 0.07, χ²/df = 2.8), and Nurses’ Counterproductive Work Behaviors (CFI = 0.97, RMSEA = 0.05, χ²/df = 2.2) all showed satisfactory model fit indices according to confirmatory factor analyses (CFA). These values validated the structural validity of the Arabic versions by meeting widely recognized parameters (CFI > 0.90, RMSEA < 0.08, χ²/df < 3).

Initially, sample adequacy was evaluated using Bartlett’s Test of Sphericity and the Kaiser-Meyer-Olkin (KMO). A significance level of 0.05 and a minimum KMO value of 0.60 were considered essential for the validity of Bartlett’s Test of Sphericity.

The results showed that the narcissistic admiration and rivalry questionnaire had a value of 0.875 (*P* < 0.001), the nurses’ vengeful behaviors scale came in at 0.910 (*P* < 0.001), and the nurses’ counterproductive work behaviors had a value of 0.925 (*P* < 0.001). Since the factor loadings for every concept this study looked at were higher than the suggested cutoff of 0.70, the construct validity of the scales was validated.

The average variance extracted (AVE) values for each dimension of the research variable also show convergent validity [35]. Convergent validity was assessed using the average variance extracted (AVE) values for every component. AVE values greater than 0.50, which demonstrate that the construction explains most of the variance, are indicative of convergent validity. By comparing the squared correlations between the constructs with the AVE values, discriminant validity was evaluated. Since each AVE value is above the squared correlations, discriminant validity was deemed to be satisfied. As a result, the measures employed in this investigation were found to possess both discriminant and convergent validity.

### Pilot study

A pilot study was overseen with 10% of the target sample (*n* = 40 nurses) to assess the clarity, simplicity, and feasibility of the data collection tools. No changes were required. Pilot participants were excluded from the main study. Survey accuracy and completeness were verified by the researchers.

### Overcame the problem of common method biases

To counteract any common method bias (CMB), the authors used a mix of statistical, procedural, and design controls. Participants’ anonymity and secrecy were specifically guaranteed to reduce social desirability bias and promote truthful responses. The questionnaire was designed with clear instructions, alternative response forms, and unique scales to further decrease the possibility that respondents would give similar answers to several topics. Harman’s single-factor test was used for statistical analysis, and the results showed that no single factor explained the bulk of the variance, indicating that CMB was not a serious worry. Furthermore, the measurement model was validated using confirmatory factor analysis (CFA), which made sure the constructs were separate and not unduly associated. To improve the items’ clarity and reduce any ambiguity that can lead to CMB, a pilot study including 10% of the sample was also conducted. The authors successfully reduced the possible influence of common method bias on the study’s conclusions by utilizing these techniques.

### Data collection

The researchers first met with the unit nurse managers to arrange access following staff schedules and break times to gather a list of all nurses and obtain permission for data collection. At a certain time, nurses who agreed to participate were given individual surveys after receiving a thorough briefing and research instructions. Before filling out the survey, each participant was given a two-minute overview of the study’s objectives. To guarantee neutrality, response clarity, and data completeness, the instruments were filled out in front of the researchers. Completing the questionnaire took 15 to 20 min on average. The two months from January to March 2025 were used for data collection. The researchers answered all participants’ questions and offered any clarifications that were required during the procedure.

### Data analysis

Data was analyzed using IBM SPSS Statistics (Version 23) for descriptive and inferential statistics, and IBM SPSS AMOS (Version 23) for structural equation modeling (SEM). Descriptive statistics (means, standard deviations, frequencies, and percentages) were used to summarize participants’ demographic characteristics and responses to the study tools.

ANOVA and independent sample t-tests were used to analyze group differences in study variables across demographic categories (e.g., age, gender, and marital status). These parametric tests were used because the data satisfied the homogeneity of variance and normality assumptions, which were evaluated using Levene’s test and Shapiro-Wilk tests, respectively.

The narcissism of nurse supervisors, nurses’ vindictive behaviors, and counterproductive work behaviors were the three primary continuous variables whose interactions were evaluated for strength and direction using Pearson’s correlation analysis. Multiple linear regression analyses were employed to investigate the predictive effects of nurses’ vindictive behaviors and nurse managers’ narcissism on unproductive work behaviors. To verify model stability, multicollinearity was examined using the variance inflation factor (VIF) values. Structural equation modeling (SEM) by AMOS was used to test the proposed mediation model. The Chi-square statistic (χ²/df), the Comparative Fit Index (CFI), the Tucker-Lewis Index (TLI), the Root Mean Square Error of Approximation (RMSEA), and the Standardized Root Mean Square Residual (SRMR) were among the several fit indices used to assess the model’s fit.

Cronbach’s alpha coefficients and composite reliability (CR) were used to evaluate the measurement tools’ dependability; values greater than 0.70 were deemed satisfactory. Each assessment tool’s construct validity and dimensional structure were evaluated using confirmatory factor analyses (CFA).

## Results

Table 1: distribution of the studied nurses according to demographic data (*n* = 400).

**Table 1:** Distribution of the studied nurses according to demographic data (*n* = 400)

Demographic characteristics	No.	%
**Age (years)**		
20–30	100	25.0
30–40	101	25.3
≥ 40	199	49.8
** Mean ± SD**	**40.7 ± 14.2**	
**Sex**		
Male	6	1.5
Female	394	98.5
**Marital status**		
Married	393	98.3
Divorced	4	1.0
Widowed	3	0.8
**Qualification**		
Technical nurse	7	1.8
Professional nurse	393	98.3
**Job position**		
Internal department	99	24.8
Others	301	75.3
**Experience year of nursing**		
5–10	99	24.8
10–15	101	25.3
> 15	200	50.0
**Mean ± SD**	**16.21 ± 7.55**	
**Experience hospital**		
5–10	112	28.0
10–15	127	31.8
> 15	161	40.3
**Mean ± SD**	**12.30 ± 8.60**	

Nearly half (49.8%) of the 400 nurses in the study were 40 years of age or older, with a mean age of 40.7 ± 14.2 years, according to their demographic data. The great majority were married (98.3%) and female (98.5%). Just 1.8% were technical nurses, compared to 98.3% who were professional nurses. Experience levels varied, with an average of 12.30 ± 8.60 years in hospitals and 16.21 ± 7.55 years in nursing. For example, 40.3% had been employed at hospitals for more than 15 years, and half (50.0%) had been nurses for more than 15 years.

Table 2: distribution of the studied nurses according to their levels and mean percent.

**Table 2:** Distribution of the studied nurses according to their levels and mean percent score of narcissism, nurses’ counterproductive work behaviors, and nurses’ vengeful behaviors (*n* = 400)

	Low		Moderate		High		Total score	Mean score	Mean percent score
	No.	%	No.	%	No.	%	Mean ± SD	Mean ± SD	Mean ± SD
Narcissistic Admiration and Rivalry Questionnaire (NARQ)	98	24.5	201	50.3	101	25.3	54.80 ± 13.86	3.04 ± 0.77	51.10 ± 19.25
**Admiration strategy**	**102**	**25.5**	**197**	**49.3**	**101**	**25.3**	**24.78 ± 10.92**	**2.75 ± 1.21**	**43.83 ± 30.34**
Grandiosity	200	50.0	99	24.8	101	25.3	8.50 ± 2.62	2.83 ± 0.87	45.81 ± 21.81
Striving for uniqueness	199	49.8	100	25.0	101	25.3	8.27 ± 4.44	2.76 ± 1.48	43.88 ± 37.0
Charming Ness	201	50.3	98	24.5	101	25.3	8.02 ± 4.43	2.67 ± 1.48	41.79 ± 36.95
**Rivalry strategy**	**0**	**0.0**	**299**	**74.8**	**101**	**25.3**	**30.02 ± 3.69**	**3.34 ± 0.41**	**58.38 ± 10.25**
Devaluation	200	50.0	200	50.0	0	0.0	7.26 ± 1.78	2.42 ± 0.59	35.46 ± 14.86
Striving for supremacy	101	25.3	200	50.0	99	24.8	11.01 ± 4.08	3.67 ± 1.36	66.71 ± 34.04
Aggressiveness	0	0.0	99	24.8	301	75.3	11.76 ± 1.64	3.92 ± 0.55	72.98 ± 13.63
**Nurses’ Counterproductive Work Behaviors**	**400**	**100.0**	**0**	**0.0**	**0**	**0.0**	**42.40 ± 5.96**	**1.23 ± 0.19**	**5.85 ± 4.76**
Sabotage	400	100.0	0	0.0	0	0.0	3.51 ± 0.87	1.17 ± 0.29	4.21 ± 7.25
Withdrawal	400	100.0	0	0.0	0	0.0	4.51 ± 0.50	1.13 ± 0.13	3.19 ± 3.13
Production deviance	400	100.0	0	0.0	0	0.0	3.79 ± 1.32	1.26 ± 0.44	6.56 ± 11.01
Theft	400	100.0	0	0.0	0	0.0	6.31 ± 2.20	1.36 ± 0.25	8.76 ± 13.50
Abuse	400	100.0	0	0.0	0	0.0	24.29 ± 4.01	1.35 ± 0.22	8.73 ± 5.57
**Nurses Vengeful Behaviors**	**66**	**16.5**	**334**	**83.5**	**0**	**0.0**	**24.73 ± 4.29**	**2.75 ± 0.48**	**43.70 ± 11.91**

SD: Standard deviation

The mean score for the Narcissistic Admiration and Rivalry, which measures the degree of narcissistic traits and counterproductive work behaviors, was 54.80 ± 13.86. Of nurses, 24.5% scored low, 50.3% moderate, and 25.3% high. Grandiosity, aiming for uniqueness, and Charming Ness subscales scored 8.50 ± 2.62, 8.27 ± 4.44, and 8.02 ± 4.43, respectively, which contributed to the adoration strategy’s mean score of 24.78 ± 10.92. With a mean score of 30.02 ± 3.69, the competition strategy scored 7.26 ± 1.78 for devaluation, 11.01 ± 4.08 for striving for supremacy, and 11.76 ± 1.64 for aggressiveness. At 42.40 ± 5.96, the mean score for all nurses was counterproductive. Abuse had the greatest mean (24.29 ± 4.01) among its subscales, followed by sabotage (3.51 ± 0.87), withdrawal (4.51 ± 0.50), production deviance (3.79 ± 1.32), and theft (6.31 ± 2.20). Additionally, with an overall mean score of 24.73 ± 4.29, 83.5% of nurses exhibited moderate levels of vengeful behaviors.

Table 3: Correlation between the studied variables.

**Table 3:** Correlation between narcissism, nurses’ counterproductive work behaviors, and nurses’ vengeful behaviors (*n* = 400)

		Grandiosity	Striving for uniqueness	Charming Ness	Admiration strategy	Devaluation	Striving for supremacy	Aggressiveness	Rivalry strategy	NARQ	Sabotage	Withdrawal	Production deviance	Theft	Abuse	Counterproductive Behaviors	Nurses Vengeful Behaviors
Grandiosity	**r**																
	**p**																
Striving for uniqueness	**r**	0.679*															
	**p**	< 0.001*															
Charming Ness	**r**	0.911*	0.917*														
	**p**	< 0.001*	< 0.001*														
**Admiration strategy**	**r**	0.886*	0.942*	0.997*													
	**p**	< 0.001*	< 0.001*	< 0.001*													
Devaluation	**r**	0.040	0.016	0.043	0.021												
	**p**	0.423	0.748	0.391	0.683												
Striving for supremacy	**r**	0.669*	0.502*	0.659*	0.632*	0.761*											
	**p**	< 0.001*	< 0.001*	< 0.001*	< 0.001*	< 0.001*											
Aggressiveness	**r**	0.199*	0.288*	0.078	0.101*	0.745*	0.488*										
	**p**	< 0.001*	< 0.001*	0.120	0.044*	< 0.001*	< 0.001*										
**Rivalry strategy**	**r**	0.633*	0.691*	0.743*	0.734*	0.689*	0.955*	0.623*									
	**p**	< 0.001*	< 0.001*	< 0.001*	< 0.001*	< 0.001*	< 0.001*	< 0.001*									
**NARQ**	**r**	0.866*	0.926*	0.984*	0.984*	0.200*	0.753*	0.245*	0.845*								
	**p**	< 0.001*	< 0.001*	< 0.001*	< 0.001*	< 0.001*	< 0.001*	< 0.001*	< 0.001*								
Sabotage	**r**	0.404*	0.115*	0.144*	0.108*	0.414*	0.105*	0.756*	0.251*	0.019							
	**p**	< 0.001*	0.021*	0.004*	0.030*	< 0.001*	0.036*	< 0.001*	< 0.001*	0.711							
Withdrawal	**r**	0.179*	0.363*	0.103*	0.146*	0.000	0.048	0.458*	0.150*	0.155*	0.593*						
	**p**	< 0.001*	< 0.001*	0.040*	0.003*	0.999	0.340	< 0.001*	0.003*	0.002*	< 0.001*						
Production deviance	**r**	0.317*	0.337*	0.371*	0.364*	0.539*	0.619*	0.454*	0.626*	0.453*	0.347*	0.585*					
	**p**	< 0.001*	< 0.001*	< 0.001*	< 0.001*	< 0.001*	< 0.001*	< 0.001*	< 0.001*	< 0.001*	< 0.001*	< 0.001*					
Theft	**r**	0.317*	0.337*	0.371*	0.364*	0.539*	0.619*	0.454*	0.626*	0.453*	0.347*	0.585*	0.990*				
	**p**	< 0.001*	< 0.001*	< 0.001*	< 0.001*	< 0.001*	< 0.001*	< 0.001*	< 0.001*	< 0.001*	< 0.001*	< 0.001*	< 0.001*				
Abuse	**r**	0.384*	0.272*	0.063	0.007	0.192*	0.314*	0.396*	0.080	0.027	0.622*	0.930*	0.255*	0.255*			
	**p**	< 0.001*	< 0.001*	0.206	0.887	< 0.001*	< 0.001*	< 0.001*	0.112	0.593	< 0.001*	< 0.001*	< 0.001*	< 0.001*			
**Counterproductive Behaviors**	**r**	0.027	0.396*	0.206*	0.238*	0.129*	0.135*	0.463*	0.292*	0.266*	0.527*	0.969*	0.761*	0.761*	0.811*		
	**p**	0.590	< 0.001*	< 0.001*	< 0.001*	0.010*	0.007*	< 0.001*	< 0.001*	< 0.001*	< 0.001*	< 0.001*	< 0.001*	< 0.001*	< 0.001*		
**Nurses Vengeful Behaviors**	**r**	0.267*	0.332*	0.316*	0.327*	0.371*	0.081	0.225*	0.010	0.255*	0.108*	0.203*	0.061	0.061	0.189*	0.196*	
	**p**	< 0.001*	< 0.001*	< 0.001*	< 0.001*	< 0.001*	0.107	< 0.001*	0.842	< 0.001*	0.032*	< 0.001*	0.222	0.222	< 0.001*	< 0.001*	

r: Pearson coefficient *: Statistically significant at *p* ≤ 0.05

Significant positive associations between narcissistic traits and unproductive work habits were found using correlation analysis. Striking for originality (*r* = 0.679, *p* < 0.001) and Charming Ness (*r* = 0.911, *p* < 0.001) were substantially associated with grandiosity, but the adoration strategy was significantly associated with Charming Ness (*r* = 0.997, *p* < 0.001). Aggression was strongly correlated with counterproductive work behaviors (*r* = 0.463, *p* < 0.001), and striving for dominance was positively connected with devaluation (*r* = 0.761, *p* < 0.001). Additionally, there was a strong correlation between nurses’ vengeful actions and counterproductive work behaviors (*r* = 0.196, *p* < 0.001).

Table 4: linear regression models examining associations with nurses’ counterproductive work behaviors.

**Table 4:** Linear regression models for factors affecting nurses’ counterproductive work behaviors

Factors	B	Beta	t	*p*	95% CI	
					LL	UL
Narcissistic Admiration and Rivalry Questionnaire (NARQ)	0.099	0.231	4.654*	< 0.001*	0.057	0.141
Nurses Vengeful Behaviors	0.191	0.137	2.768*	< 0.001*	0.055	0.326

R^2^ = 0.088, Adjusted R^2^ = 0.084, F = 19.199*, *p* < 0.001^*^

F, p: f and p values for the model

R^2^: Coefficient of determination

B: Unstandardized Coefficients

Beta: Standardized Coefficients

t: t-test of significance

CI: Confidence interval

LL: Lower limit

UL: Upper Limit

*: Statistically significant at *p* ≤ 0.05

The correlation between the narcissistic qualities of nurse managers, the spiteful actions of nurses, and the counterproductive work behaviors (CWBs) of nurses was investigated using linear regression analysis. The findings showed that nurses’ vindictive behaviors (B = 0.191, Beta = 0.137, t = 2.768, *p* < 0.001) and narcissistic admiration and rivalry (B = 0.099, Beta = 0.231, t = 4.654, *p* < 0.001) were both strongly correlated with CWBs. The corrected R2 = 0.084 (F = 19.199, *p* < 0.001) showed that the model explained around 8.4% of the variance in CWBs.

**Fig. 2:**
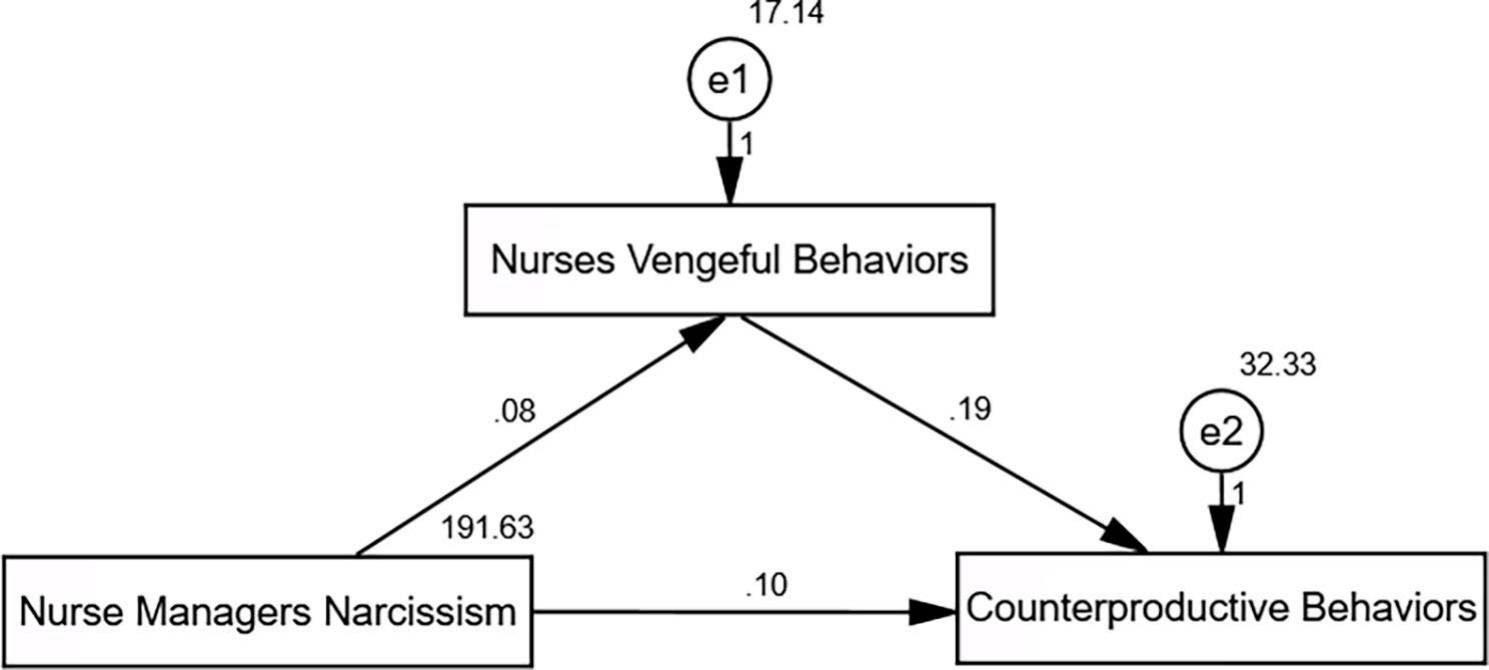
Path analysis of the direct and indirect effect of Nurse Managers’ Narcissism on Nurses’ Counterproductive Work Behaviors mediating by Nurses’ Vengeful Behaviors

Table 5; Fig. 2: statistical mediation of the association between nurse managers’ narcissism and nurses’ counterproductive work behaviors by nurses’ vengeful behaviors.

**Table 5:** The direct and indirect effect of the mediating role of nurses’ vengeful behaviors in the link between nurse managers’ narcissism and nurses’ counterproductive work behaviors

			Direct effect	Indirect effect	Estimate	S.E.	C.*R*.	*P*
Counterproductive Work Behaviors	<---	Nurses’ Vengeful Behaviors	0.191		0.137	0.069	2.775*	0.006*
Nurses’ Vengeful Behaviors	<---	Nurse Managers’ Narcissism	0.079		0.255	0.015	5.276*	< 0.001*
Counterproductive Work Behaviors	<---	Nurse Managers’ Narcissism	0.099	0.015	0.231	0.021	4.665*	< 0.001*

Model fit parameters CFI; IFI; RMSEA (1.000; 1.000; 0.079)

Model χ^2^/df. 21.248/3 *p* ≤ 0.001

CFI: Comparative Fit Index, IFI: Incremental Fit Index, RMSEA: Root Mean Square Error of Approximation

The statistical mediation of the association between the narcissism of nurse managers and the unproductive work behaviors of nurses through the vengeful behaviors of nurses was investigated using path analysis. CWBs and nurses’ vindictive actions were found to be significantly correlated by the analysis (estimate = 0.191, S.E. = 0.069, C.R. = 2.775, *p* = 0.006). Furthermore, there was a statistically significant correlation between nurse managers’ narcissism and vindictive actions (estimate = 0.079, S.E. = 0.015, C.R. = 5.276, *p* < 0.001). These findings lend credence to a statistical mediation model, suggesting that the association between nurses’ CWBs and nurse managers’ narcissistic qualities may be explained in part by nurses’ vindictive actions. The suggested model fit the data well, according to model fit indices (CFI = 1.000, IFI = 1.000, RMSEA = 0.079, χ²/df = 21.248/3, *p* ≤ 0.001).

## Discussion

Nurse managers can be self-serving, arrogant, aggressive, and incompetent, even though they are typically seen as knowledgeable, experienced, and moral in their actions [28]. All these are characteristics of narcissistic behavior. Narcissistic nurse managers have leadership philosophies and practices that are typically driven by their desire for recognition and power rather than a genuine concern for the nurses and healthcare organizations they oversee [16]. So, this study aims to explore the relationship between nurse managers’ narcissism and nurses’ counterproductive work behaviors and the mediating role of nurses’ vengeful behaviors.

### Distribution of the studied nurses according to demographic data

This study revealed that nurses with more experience and age may be more sensitive to interpersonal dynamics and less accepting of harmful management styles, particularly when those styles jeopardize workplace harmony or professional ideals. Additionally, high marriage rates could influence how nurses handle stress and conflict at work by fostering emotional resilience or external support networks. However, having a lot of family obligations can also lead to additional emotional stress, which increases the impact of hostile work situations. Furthermore, according to the highly professional sample, everyone is knowledgeable of patient-centered care and ethical norms. Retaliatory attitudes may be stoked by exposure to narcissistic leadership, which is perceived as a more serious breach of professional values.

### Distribution of the studied nurses according to their levels and mean percent

The results of this study provide important light on how common narcissistic tendencies and counterproductive work practices are among nurses. A moderate level of narcissistic tendencies is shown by the nursing sample’s mean score of 54.80 ± 13.86 on the Narcissistic Admiration and Rivalry scale. Interestingly, about half (50.3%) of the nurses had moderate narcissism, with roughly equal percentages scoring high (25.3%) and low (24.5%). These results suggest that to preserve their self-image, nurses believe this may be due to the manager capturing their attention in conversation most of the time and they are deserving of praise for their amazing personalities. These results are consistent with Abd-Allah and Sleem (2024), which showed the overall narcissistic leadership level at Main Mansoura University Hospital and Mansoura General Hospital was primarily moderate (43.8% and 58.9%, respectively [42]. Moreover, according to Ghislieri, Cortese, Molino, and Gatti’s (2019) research, nurses at a hospital in the northwest of Italy exhibited a moderate degree of narcissistic leadership because they feel like exceptional individuals who thrive on praise and achievements [43].

The mean score of 42.40 ± 5.96 for counterproductive work habits indicates that nurses have a considerable prevalence of unfavorable workplace behaviors. Verbal hostility, mistreatment, and other unpleasant interactions, such as neglecting another employee, accusing them of a mistake, and verbally insulting them at work, may be common in nursing settings, according to the most significant subscale, abuse (24.29 ± 4.01). Abusive behaviors might worsen stress and exhaustion by fostering a toxic work environment. This result is contrary to Elliethey et al. (2024), who revealed the majority of nurses reporting low levels of counterproductive work behaviors (82.25%) [21].

Furthermore, the prevalence of vengeful behaviors was noteworthy, with an overall mean score of 24.73 ± 4.29. Retaliatory behavior can be a common response to perceived injustices and narcissistic management at work, as seen by the noteworthy finding that 83.5% of nurses had moderate degrees of vindictive impulses. This study highlights the importance of organizational activities in fostering a supportive and cooperative work environment and lowering the probability of conflict and hostility at work.

### Table (3): correlation between the studied variables

By emphasizing the strong positive associations between narcissistic traits and poor work habits, the current study supports the hypothesis that managers with higher levels of narcissism may engage in counterproductive work practices. Specifically, grandiosity was significantly associated with Charming Ness (*r* = 0.911, *p* < 0.001) and striking for originality (*r* = 0.679, *p* < 0.001), according to the correlation study. These studies suggest that individuals who wish to make a statement by being distinctive and endearing may do so in a way that feeds their inflated sense of self. This is also because narcissistic managers commonly utilize their behavior to try to attract attention and praise, which leads to counterproductive behaviors from subordinated employees.

Furthermore, the strong association between charming Ness and adoring approach (*r* = 0.997, *p* < 0.001) implies that those who employ adoration as a social strategy may be particularly adept at using charm to persuade or attract others. Narcissistic individuals employ interpersonal strategies to maintain their social standing and self-image. These results are consistent with Asif et al. (2024), who revealed that narcissism significantly influences unproductive job behavior both directly and indirectly, especially when there are work-related stressors present. Additionally, this study revealed that one of the most unpleasant situations in today’s workplace is counterproductive work conduct [44].

The results also indicate a high link between aggression and unproductive work practices (*r* = 0.463, *p* < 0.001). This suggests that those who are more aggressive are more likely to act in ways that jeopardize harmony and efficiency at work. This finding demonstrates that aggressive tendencies are a defining feature of negative workplace behaviors like hostility, bullying, and sabotage.

Additionally, a strong link was found between the desire for dominance and devaluation (*r* = 0.761, *p* < 0.001), indicating that those who strive for dominance may also act in ways that disparage or reduce others. This finding demonstrates the narcissistic tendency to utilize social manipulation and dominance-seeking strategies to maintain supremacy and finally lead to counterproductive behaviors from subordinates.

The study also found a strong association between counterproductive work practices and vengeful actions (*r* = 0.196, *p* < 0.001). This link suggests that individuals with spiteful tendencies may employ retaliatory behaviors to exacerbate workplace dysfunction, even if it isn’t as strong as the others. This indicates that when an employee feels that their position or sense of value is under jeopardy, they are more likely to act in ways that seek retaliation.

### Linear regression models examining associations with nurses’ counterproductive work behaviors

The study’s findings demonstrate the strong correlations between nurses’ counterproductive work behaviors (CWBs), narcissistic admiration and rivalry (NAR), and vindictive actions. According to linear regression analysis, spiteful behaviors (B = 0.191, Beta = 0.137, t = 2.768, *p* < 0.001) and NAR (B = 0.099, Beta = 0.231, t = 4.654, *p* < 0.001) were both substantially correlated with CWBs. According to these results, those who exhibit more narcissistic traits, especially those that include rivalry and admiration-seeking, may also be more likely to engage in actions that reduce productivity at work. Like this, nurses who report having spiteful inclinations are also more likely to report engaging in ineffective behaviors, which may be a response to interpersonal conflict or feelings of unfairness at work.

Counterproductive work behaviors (CWB) were found to be significantly correlated with vengeful and narcissistic admiration and rivalry (NAR) behaviors. However, the model’s adjusted R2 of 0.084 suggests that these factors only account for 8.4% of the variance in CWBs, indicating that other factors also play a role in these workplace behaviors. The combination of retaliatory inclinations and personality traits is significantly linked to workplace dynamics, according to the statistically significant overall model (F = 19.199, *p* < 0.001). These results are consistent with earlier studies that found links between narcissistic traits and misconduct at work, especially in settings with strict hierarchical structures and significant interpersonal stress [16].

In the nursing profession, where cooperation, emotional fortitude, and moral behavior are essential, the existence of narcissistic rivalry and vindictive actions may be linked to weakened team cohesion and possibly poor patient care. Nonetheless, there are conflicting results in the literature. For example, Davison et al. (2022) found no significant correlation between narcissism and either type of workplace misbehavior (*p* = 0.10), indicating that the influence of narcissistic traits may vary depending on the situation. Zhang and Cui (2024), on the other hand, discovered that narcissism was positively correlated with creative self-efficacy (β = 0.42, *p* < 0.001) and creative deviance (β = 0.64, *p* < 0.001), with the link being partially mediated by creative self-efficacy. Their findings also showed that, in the presence of strong leadership, employee narcissism and creative deviance were positively correlated. These conflicting results highlight how complicated narcissism’s function in organizational behavior is and imply that its effects might change based on contextual and situational circumstances [45].

### Statistical mediation of the association between nurse managers’ narcissism and nurses’ counterproductive work behaviors by nurses’ vengeful behaviors

The study’s findings shed light on the relationships between the narcissistic qualities of nurse managers and the behaviors of nurses in the workplace, namely retaliatory acts and counterproductive work behaviors (CWBs). The results imply that more frequent reports of vindictive behaviors among nurses are linked to higher levels of narcissism in nurse managers, and that these reports are linked to increased participation in CWBs. These correlations point to the possible behavioral and relational issues that could occur in settings where narcissistic leadership traits are viewed, with potential ramifications for both corporate and employee well-being.

One of the study’s main conclusions is that nurses’ counterproductive work behaviors (CWBs) through vindictive behaviors are statistically mediated by the narcissistic features of nurse managers. Vengeful behaviors and CWBs were shown to be substantially correlated (estimate = 0.191, *p* = 0.006). This suggests that nurses may be more prone to report retaliatory inclinations that co-occur with increased CWBs in settings where narcissistic leadership traits are recognized. Furthermore, the theoretical claim that perceived leadership traits are connected to employee behavioral responses is supported by the indirect relationship between perceived management narcissism and vindictive behaviors (estimate = 0.079, *p* < 0.001). These results highlight the importance of looking at interpersonal dynamics in influencing behavior at work, even though they are not causative.

The proposed statistical mediation model fits the data well, supporting the hypothesized relationships between managerial narcissism, vengeful behaviors, and counterproductive work behaviors (CWBs), according to strong model fit indices (CFI = 1.000, IFI = 1.000, RMSEA = 0.079, χ²/df = 21.248/3, *p* ≤ 0.001). These results support previous studies on workplace deviance and destructive leadership, indicating a potential connection between dysfunctional workplace dynamics and narcissistic leadership qualities. The cross-sectional nature of the study limits drawing inferences about causality; thus, even while the results show statistically significant connections, they should be regarded with caution.

Practically speaking, these results imply that healthcare institutions can gain from keeping a close eye on and resolving nurse managers’ narcissistic characteristics to lessen the likelihood that they will be linked to unfavorable workplace behaviors. Initiatives for leadership development that emphasize empathy, emotional intelligence, and moral judgment may help to lessen the manifestation of narcissistic qualities in managerial positions. Furthermore, giving nurses access to structured conflict resolution techniques and psychological support networks may enable them to handle difficult leadership situations without turning to vindictive or counterproductive actions. Future studies should investigate potential moderating factors that could affect the direction or degree of these correlations, such as individual resilience, corporate culture, and team cohesion. To learn more about the temporal dynamics and possible long-term impacts of narcissistic leadership on worker well-being and the caliber of healthcare services, longitudinal studies are also advised.

## Conclusion

This study emphasizes the associations between nurses’ workplace actions and management narcissism, highlighting the mediation function of vengeful tendencies in the relationship between unproductive work behaviors and perceived narcissistic features in nurse supervisors. These results highlight the value of developing leadership styles that promote a morally sound and psychologically sound workplace. In addition to improving organizational productivity and employee happiness, addressing dysfunctional leadership styles may help reduce workplace deviance. The findings imply that retaliatory actions could be a major mechanism by which nursing staff members’ opinions of narcissistic management are connected to ineffective actions.

Hospital managers can think about encouraging open communication between nursing leaders and personnel to lessen the possible unfavorable workplace dynamics linked to managerial narcissism. A more collaborative and productive work environment may result from promoting nurses’ involvement in committee meetings and group decision-making, both in-person and virtually. In addition to fostering a culture of respect and shared responsibility, these tactics may help ease interpersonal conflicts [46–48].

### Implications for nursing practice and policy

The study’s conclusions provide significant new information for nursing practice and healthcare policy. The necessity for focused solutions is highlighted by the realization that the narcissistic characteristics of nurse managers can lead to a poisonous workplace that encourages retaliatory and ineffective actions from nursing staff. Programs for developing nurse leaders’ self-awareness, emotional intelligence, and ethical leadership should be given top priority by healthcare organizations. Incorporating coaching and psychological screening into managerial positions can also lessen the negative consequences of narcissistic tendencies. Organizational culture can be enhanced at the policy level by enforcing zero-tolerance rules for abusive or retaliatory leadership practices and encouraging open, encouraging lines of communication.

Clinically speaking, creating a happy work atmosphere is probably going to improve nurse retention, job happiness, and, eventually, patient safety and the standard of care. The creation of institutional wellness initiatives that tackle workplace pressures and enable nurses to report toxic behaviors without worrying about reprisals is also supported by these findings.

### Strengths and limitations

This study’s utilization of a thorough strategy to comprehend the connection between nurse managers’ actions and nurses’ job outcomes is one of its main advantages. The study offers a sophisticated knowledge of how organizational culture and leadership qualities affect nurses’ behaviors by combining descriptive statistics, correlation analysis, regression modeling, and structural equation modeling. The findings’ robustness and generalizability are further improved by the large sample size and the inclusion of numerous demographic variables. Additionally, the study’s measurements are strengthened, and the correctness of the constructs involved is guaranteed using various reliability measures, such as Cronbach’s alpha and composite reliability.

Several limitations should be acknowledged when evaluating the conclusions of this study. First, the capacity to make causal conclusions is constrained by the cross-sectional, correlational design. These findings show associational patterns rather than clear-cut causal pathways, notwithstanding the statistical mediation analyses that were performed. Specifically, it is impossible to verify the temporal order of the narcissistic qualities of nurse managers, the spiteful actions of nurses, and the counterproductive work behaviors (CWBs). It’s still feasible that unmeasured third variables have an impact or that the interactions are bidirectional. Establishing causality and elucidating the directionality of the observed relationships requires experimental or longitudinal research methodologies. Despite efforts to assure anonymity and minimize response bias, the use of self-reported measurements may nonetheless introduce common method bias. Also, the results’ applicability outside of this particular hospital setting is restricted by the convenience sample’s use from a single institution with a nursing population that is primarily married, female, and professionally homogeneous.

### Future studies

To better understand causality and the long-term consequences of leadership styles, future research should examine the longitudinal effects of nurse managers’ behaviors on nurses’ work outcomes. Future studies should also look at how alternative leadership philosophies, including transformational or servant leadership, affect nurses’ unproductive work practices and the factors that drive them. The results would be more broadly applicable if the sample was expanded to encompass a wider variety of healthcare environments, such as various nations or regions. The relationship between leadership behaviors and nurses’ work outcomes could also be examined in terms of organizational and individual aspects, such as stress, job satisfaction, and support networks. Lastly, intervention studies that evaluate particular tactics to enhance the leadership abilities of nurse managers and lessen unfavorable behaviors may offer insightful information for enhancing nursing practice and workplace culture.

## Data Availability

The datasets developed and examined for this investigation are available from the corresponding author upon reasonable request.
